# Supplementary material for: Re-examination of the risk of autoimmune diseases after dengue virus infection: A population-based cohort study
Source: PLoS Negl Trop Dis. 2023 Mar 7;17(3):e0011127. doi: 10.1371/journal.pntd.0011127 (PMC9990932; doi:10.1371/journal.pntd.0011127)
Supplement: S1 Table — (DOCX) [file pntd.0011127.s001.docx]

**S1 Table. List of ICD codes for identifying diseases in this study**

|  | **ICD-9-CM (until 2015)** | **ICD-10-CM (since 2016)** |
| --- | --- | --- |
| **Autoimmune diseases** |  |  |
| **Catastrophic illness** |  |  |
| Systemic lupus erythematosus | 710.0 | M32 |
| Systemic sclerosis | 710.1 | M34 |
| Sjogren's syndrome | 710.2 | M35.0 |
| Inflammatory myopathy | 710.3, 710.4 | M33.0, M33.1, M33.9, M36.0, M33.2 |
| Rheumatoid arthritis | 714.0, 714.30-714.33 | M05.7-M06.0, M06.2, M06.3, M06.8, M06.9, M08 |
| Behcet's syndrome | 136.1 | M35.2 |
| Systemic vasculitis | 443.1, 446.0, 466.2, 446.4, 446.5, 446.7, 446.1 | I73.1, M30.0, M30.2, M30.8, M31.0, M31.3, M31.5, M31.6, M31.4, M30.3 |
| Type I DM | 250.x1, 250.x3 | E10.1~E10.9 |
| Multiple sclerosis | 340 | G35 |
| Myasthenia gravis | 358.0 | G70.0 |
| Inflammatory bowel diseases | 555, 556 | K50, K51 |
| Autoimmune hemolytic anemia | 283.0 | D59.0, D59.1 |
| Pemphigus | 694.4 | L10 |
| **Non-catastrophic illness** |  |  |
| Ankylosing spondylitis | 720.0 | M08.1, M45 |
| Post-infectious arthritis | 711.1, 099.3 | M02.3 |
| Uveitis | 364.00, 364.01, 364.3 | H20.00, H20.01, H20.9 |
| Psoriasis | 696.0, 696.1 | L40 |
| Autoimmune thyroid disease | 242.0, 245.2 | E05.0, E06.3 |
| Primary adrenocortical insufﬁciency | 255.4 | E27.1, E27.2 |
| Guillain–Barré syndrome | 357.0 | G61.0 |
| Autoimmune encephalomyelitis | 323.6, 323.8, 323.9 | G04.00, G04.01, G04.30, G04.31, G04.39, G04.81, G04.89, G04.90, G04.91, G05.4, G37.3, G37.4 |
| Celiac disease | 579.0 | K90.0 |
| **Infectious diseases** |  |  |
| HIV | 042-044 |  |
| TB | 010-018 |  |
| Bacterial infection | 001-005, 008.1-008.5, 020-027, 030-041, 076, 320, 420.9, 421.0, 422.92, 4 81-4 83, 511.1, 522.4-522.7, 523.3-523.5, 527.3, 528.3, 566, 567.0-567.2, 569.5, 572.0, 590, 595.89, 595.9, 597.0, 599.0, 614-616, 680-686, 785.52 |  |
| Viral infection | 008.6, 008.8, 045-049, 050-059, 060-066, 070-075, 077-079, 321.2, 323.0, 422.0, 460, 466, 480, 487 |  |
| Other infection | 006-007, 009, 080-139, 321.0, 321.1, 321.3, 321.4, 321.8, 323.1, 323.2, 323.4, 324, 415.12, 420.0, 421.1, 421.9, 461-465, 475, 478.20-478.24, 484-486, 510, 511.0, 513, 601, 604 |  |
| **Comorbidities** |  |  |
| Cerebrovascular accident | 430-438 |  |
| COPD | 491-494, 496 |  |
| DM | 250 |  |
| Dyslipidaemia | 272 |  |
| Hypertension | 401-405 |  |
| Ischaemic heart disease | 410-414 |  |
| Liver cirrhosis | 570, 571.2, 571.5, 571.6 |  |
| Malignancy | 140-208 |  |
| Renal failure | 584-586 |  |

* The National Health Insurance (NHI) program in Taiwan began to adopt ICD-10-CM in 2016.
