# Supplementary material for: Re-examination of the risk of autoimmune diseases after dengue virus infection: A population-based cohort study
Source: PLoS Negl Trop Dis. 2023 Mar 7;17(3):e0011127. doi: 10.1371/journal.pntd.0011127 (PMC9990932; doi:10.1371/journal.pntd.0011127)
Supplement: S2 Table — (DOCX) [file pntd.0011127.s002.docx]

**S2 Table. Incidence (per 10,000) of autoimmune diseases between the hospitalized dengue cases and control groups**

| Autoimmune disease | Dengue group | | | | | | Control group | | | | | |
| --- | --- | --- | --- | --- | --- | --- | --- | --- | --- | --- | --- | --- |
|  | Total  (n = 23145) | | Male  (n = 11507) | | Female  (n = 11638) | | Total  (n = 92580) | | Male  (n = 46028) | | Female  (n = 46552) | |
|  | n | IR | n | IR | n | IR | n | IR | n | IR | n | IR |
| All | 386 | 30.84 | 167 | 27.16 | 219 | 34.40 | 1322 | 26.33 | 570 | 23.12 | 752 | 29.42 |
| Autoimmune thyroid disease | 96 | 7.59 | 27 | 4.35 | 69 | 10.71 | 325 | 6.41 | 75 | 3.01 | 250 | 9.69 |
| Uveitis | 87 | 6.87 | 43 | 6.92 | 44 | 6.82 | 281 | 5.54 | 128 | 5.15 | 153 | 5.91 |
| Psoriasis | 53 | 4.18 | 28 | 4.51 | 25 | 3.87 | 235 | 4.63 | 157 | 6.32 | 78 | 3.01 |
| Primary adrenocortical insufficiency | 55 | 4.34 | 26 | 4.18 | 29 | 4.49 | 165 | 3.25 | 84 | 3.38 | 81 | 3.13 |
| Ankylosing spondylitis | 28 | 2.21 | 18 | 2.90 | 10 | 1.54 | 127 | 2.50 | 74 | 2.97 | 53 | 2.05 |
| Autoimmune encephalomyelitis | 32 | 2.52 | 16 | 2.57 | 16 | 2.47 | 32 | 0.63 | 18 | 0.72 | 14 | 0.54 |
| Rheumatoid arthritis | 13 | 1.02 | 5 | 0.80 | 8 | 1.24 | 69 | 1.36 | 11 | 0.44 | 58 | 2.24 |
| Sjögren's syndrome | 9 | 0.71 | ^b^ | ^b^ | ^b^ | ^b^ | 50 | 0.98 | ^b^ | ^b^ | ^b^ | ^b^ |
| Systemic lupus erythematosus | 6 | 0.47 | 3 | 0.48 | 3 | 0.46 | 11 | 0.22 | ^b^ | ^b^ | ^b^ | ^b^ |
| Myasthenia gravis | 5 | 0.39 | ^b^ | ^b^ | ^b^ | ^b^ | 17 | 0.33 | 9 | 0.36 | 8 | 0.31 |
| Guillain–Barré syndrome | 5 | 0.39 | ^b^ | ^b^ | ^b^ | ^b^ | 15 | 0.30 | 7 | 0.28 | 8 | 0.31 |

Abbreviation: IR, incidence rate per 10,000 person-years.

^b^ The cells left blank indicated that the number of events was less than three and were therefore not allowed to be exported under the regulations of the Health and Welfare Data Science Center of Taiwan to prevent re-identification.

*Data on Type I DM, systemic sclerosis, inflammatory myopathy, Behcet's syndrome, systemic vasculitis, multiple sclerosis, autoimmune hemolytic anemia, inflammatory myopathy, pemphigus, celiac disease, inflammatory bowel diseases and post-infectious arthritis cases were not shown because case numbers were less than three.
