# Supplementary material for: Re-examination of the risk of autoimmune diseases after dengue virus infection: A population-based cohort study
Source: PLoS Negl Trop Dis. 2023 Mar 7;17(3):e0011127. doi: 10.1371/journal.pntd.0011127 (PMC9990932; doi:10.1371/journal.pntd.0011127)
Supplement: S3 Table — (DOCX) [file pntd.0011127.s003.docx]

**S3 Table Risk of autoimmune diseases among hospitalized dengue cases after adjusting for competing mortality.**

| Autoimmune disease | Total |  | Male |  | Female |  |
| --- | --- | --- | --- | --- | --- | --- |
|  | aHR^a^ (95% CI)^b^ | p value^c^ | aHR^a^ (95% CI)^b^ | p value^c^ | aHR^a^ (95% CI)^b^ | p value^c^ |
| All | 1.14 (1.01-1.28) | 0.0302 | 1.12 (0.94-1.34) | 0.1949 | 1.15 (0.98-1.34) | 0.0868 |
| Autoimmune thyroid disease | 1.15 (0.91-1.46) | 0.2352 | 1.41 (0.89-2.25) | 0.1428 | 1.08 (0.82-1.42) | 0.5935 |
| Uveitis | 1.14 (0.90-1.46) | 0.2805 | 1.20 (0.85-1.70) | 0.2928 | 1.08 (0.77-1.52) | 0.6586 |
| Psoriasis | 0.87 (0.64-1.18) | 0.3733 | 0.66 (0.44-1.00) | 0.0484 | 1.33 (0.83-2.14) | 0.2337 |
| Primary adrenocortical insufficiency | 1.33 (0.97-1.82) | 0.0794 | 1.30 (0.83-2.05) | 0.2571 | 1.36 (0.88-2.11) | 0.1642 |
| Ankylosing spondylitis | 0.88 (0.59-1.33) | 0.5445 | 1.02 (0.61-1.70) | 0.9476 | 0.73 (0.37-1.42) | 0.3489 |
| Autoimmune encephalomyelitis | 3.66 (2.19-6.14) | <0.0001 | 3.04 (1.53-6.03) | 0.0015 | 4.85 (2.18-10.80) | 0.0001 |
| Rheumatoid arthritis | 0.77 (0.42-1.40) | 0.3899 | 1.97 (0.62-6.30) | 0.2508 | 0.56 (0.26-1.17) | 0.1223 |
| Sjögren's syndrome | 0.76 (0.36-1.60) | 0.4719 | 2.93 (0.26-33.41) | 0.3863 | 0.70 (0.32-1.52) | 0.3669 |
| Systemic lupus erythematosus | 2.91 (1.05-8.09) | 0.0404 | 7.20 (0.94-54.95) | 0.0570 | 1.82 (0.49-6.75) | 0.3705 |
| Myasthenia gravis | 1.14 (0.42-3.09) | 0.7945 | 0.89 (0.20-3.88) | 0.8725 | 1.60 (0.40-6.37) | 0.5084 |
| Guillain–Barré syndrome | 1.60 (0.60-4.27) | 0.3480 | 1.42 (0.33-6.15) | 0.6403 | 1.95 (0.51-7.49) | 0.3314 |

Abbreviations: aHR, adjusted hazard ratio; CI, confidence interval.

^a^ Adjusted Cox proportional hazard model by sex, age, area of residence, income, urbanization, hypertension, diabetes mellitus, dyslipidemia, COPD, cerebrovascular accident, renal failure, liver cirrhosis, ischaemic heart disease, and malignancy.

^b^ 95% CIs were not adjusted for multiple comparisons and thus cannot be directly used for hypothesis testing or inference.
