# Supplementary material for: Re-examination of the risk of autoimmune diseases after dengue virus infection: A population-based cohort study
Source: PLoS Negl Trop Dis. 2023 Mar 7;17(3):e0011127. doi: 10.1371/journal.pntd.0011127 (PMC9990932; doi:10.1371/journal.pntd.0011127)
Supplement: Attachment — Submitted filename: reply1015-chien_RC_1127-chien.docx [file pntd.0011127.s004.docx]

**Reply to Reviewers**

Dr. Haralampos M. Moutsopoulos	November 27th,2022

Dr. Guilherme S. Ribeiro

PLOS Neglected Tropical Diseases submission

RE: Decision on your PLOS Neglected Tropical Diseases submission (PNTD-D-22-00980) - [EMID:bec34b63d8c5f2b6]

Dear Dr. Moutsopoulos

Thank you for giving us the opportunity to respond to the reviewers’ comments regarding our above-mentioned manuscript. Each of the concerns raised by the reviewers’ has been clarified and addressed as well as incorporated in our revised manuscript. The following details the major changes made in response to the reviewers’ suggestions. All major changes made appear in red color in the revised manuscript.

**Reviewer’s Responses to Questions**

**Methods**
Reviewer #1: This retrospective cohort study using laboratory-confirmed dengue cases evaluated the risk of autoimmune diseases after DENV infection. It was found that dengue was associated with an increased short-term risk of a rare disorder, “autoimmune” encephalomyelitis, but not with other autoimmune diseases.
In the Author Summary the sentence “Autoantibodies produced during dengue infection can cross-react with several self-antigens…..” should change to: “Antibodies produced during dengue infection can cross-react with several self-antigens…..”
In the Introduction 2nd paragraph line 7-8 “autoimmune markers”, such as antinuclear antibody (ANA), immune complex (IC), and C-reactive protein (CRP)” IC and CRP  are not autoimmune markers,  ICs can be found in many chronic infectious diseases and CRP is an indicator of inflammation.

The term Reiter’s syndrome has to be changed to “post infectious arthritis”.

**Response**: Thanks for the reviewer’s suggestion. The following sentences have been modified according to the reviewer’s recommendations in the revised manuscript.

1) “Antibodies produced during dengue infection can cross-react with several self-antigens…..” (Line 56).
2) “Although this autoimmunity has been considered transient, a small follow-up study in Cuba showed that over half of dengue patients suffered from persistent dengue symptoms in the two years following infections, and commonly showed altered autoimmune markers, such as antinuclear antibody.” (Line 78).
3) “Reiter’s syndrome” has been changed to “post-infectious arthritis” (Line 86, Line 152, Line 267, Tabe 2, Table 3, and Supplementary Table 1).

Reviewer #2: This is a well written study based on health records of dengue patients in Taiwan. The objectives are clearly articulated and the study design is appropriate. A strength of the study is the very large sample size. Correct statical analyses support the conclusion and indeed are essential to reach the conclusions. There are no ethical or regulatory concerns.
**Response**: Thanks very much for the reviewer’s compliment.

--------------------

**Results**
Reviewer #1: s not clear to reviewer on what basis the authors use the term autoimmune encephalomyelitis for the syndrome following the DENV infection; did they tested the patients’ sera for specific autoantibodies? Also the length of encephalomyelitis symptoms , the severity of the disorder and the therapeutic interventions  to combat the encephalomyelitis should be presented .
The term Reiter’s syndrome should be  changed to “post infectious arthritis”

**Response**: Thanks for the reviewer’s comment. This study used claims data from the National Health Insurance (NHI) program in Taiwan, not medical charts; therefore, detailed clinical information such as duration of symptoms, severity, laboratory results, and therapeutic interventions was not available. As done by Li *et al.*, we identified cases of autoimmune diseases based on the International Classification of Diseases (ICD) codes (ICD-9-CM and ICD-10-CM). In Taiwan, people with several autoimmune diseases can apply for catastrophic illness certification to receive a copayment exemption from the NHI program, and all the applications are reviewed by rheumatologists. These autoimmune diseases, including SLE, systemic sclerosis, Sjögren’s syndrome, inflammatory myopathy, rheumatoid arthritis, Behcet’s syndrome, systemic vasculitis, type I diabetes mellitus, multiple sclerosis, myasthenia gravis, inflammatory bowel diseases, autoimmune hemolytic anemia, and pemphigus, were identified from the Registry for Catastrophic Illness, and thus the diagnoses should be 100% valid. However, for autoimmune diseases not considered as catastrophic illnesses in Taiwan, such as autoimmune encephalomyelitis, the diagnosis might be less valid. To increase the accuracy of the diagnosis, we used hospital admission or at least three outpatient visits with relevant ICD-9-CM or ICD-10-CM codes to identify cases. Although the validity was unknown, we believed that the diagnosis should be relatively accurate because the diagnosis of autoimmune encephalomyelitis in Taiwan was usually made by neurologists, infectious disease specialists or rheumatologists. We have added the following sentences in the limitation section in the revised manuscript, Lines 347-351.

“Second, the data in NHIRD were originally collected for reimbursement purposes; therefore, information on some potential confounders that might contribute to autoimmune diseases, such as smoking habits, body weight, family history, chemical exposures, and genetics, was not available. Detailed clinical information such as duration of symptoms, severity, laboratory results and therapeutic interventions was also unavailable. Third, although the diagnosis of autoimmune diseases identified from the Registry for Catastrophic Illness should be 100% valid, the diagnosis of other autoimmune diseases might be less valid. To increase the accuracy of the diagnosis, we used hospital admission or at least three outpatient visits with relevant ICD-9-CM or ICD-10-CM codes to identify cases. We believed that the diagnosis of autoimmune encephalomyelitis should be relatively accurate because this diagnosis was usually made by neurologists, infectious disease specialists or rheumatologists in Taiwan; however, the validity of remaining autoimmune diseases was less certain.”

Reviewer #2: The results are clearly presented. Dengue patients had a slightly higher risk of overall autoimmune diseases than non-dengue controls (aHR 1.16; P =0.0002). Only autoimmune encephalomyelitis remained statistically significant after Bonferroni correction for multiple testing (aHR 2.72; P < 0.0001). All the Table are presently clearly.
**Response**: Thanks very much for the reviewer’s compliment.

**Conclusions**

Reviewer #1: AS ABOVE
Reviewer #2: The conclusion of the study are justified and limitations are described appropriately. The discussion is appropriate and the public health relevance is addressed appropriately.
**Response**: Thanks very much for the reviewer’s compliment.
--------------------

**Editorial and Data Presentation Modifications**

Reviewer #1: As Above

Reviewer #2: The results are different than a previous smaller study that identified several autoimmune disease that were increased in hospitalized dengue patients. The authors do a good job of describing the differences in the studies, bit I think imless they did the sub-analysis of only hospitalized patients they should explicitly state that hospitalization might mean more severe dengue  and that more severe dengue might in fact lead to autoimmune disease.
**Response**: Thanks very much for the reviewer’s comments. We did the sub-analysis of hospitalized dengue patients only as suggested by the reviewer, which also revealed that dengue was associated with autoimmune encephalitis, but not with other autoimmune diseases (see below Tables). The association between dengue and overall autoimmune diseases became non-significant, probably because of smaller sample size (only 23145 hospitalized dengue cases and 92580 controls). We did not put this sub-analysis in the main results in the revised manuscript because this might cause more multiple testing problems. Instead, we added the following sentences in the discussion for readers who might be interested in the revised manuscript, Lines 287-291.

“Third, our study included inpatient and outpatient dengue cases, while theirs only included hospitalized patients. However, we performed subgroup analyses of hospitalized dengue cases, revealing that dengue was only associated with autoimmune encephalitis but not other autoimmune diseases (Supplementary Tables 2 and 3).”

**Supplementary Table 2. Incidence (per 10,000) of autoimmune diseases between the hospitalized dengue cases and control groups**

| Autoimmune disease | Dengue group | | | | | | Control group | | | | | |
| --- | --- | --- | --- | --- | --- | --- | --- | --- | --- | --- | --- | --- |
|  | Total  (n = 23145) | | Male  (n = 11507) | | Female  (n = 11638) | | Total  (n = 92580) | | Male  (n = 46028) | | Female  (n = 46552) | |
|  | n | IR | n | IR | n | IR | n | IR | n | IR | n | IR |
| All | 386 | 30.84 | 167 | 27.16 | 219 | 34.40 | 1322 | 26.33 | 570 | 23.12 | 752 | 29.42 |
| Autoimmune thyroid disease | 96 | 7.59 | 27 | 4.35 | 69 | 10.71 | 325 | 6.41 | 75 | 3.01 | 250 | 9.69 |
| Uveitis | 87 | 6.87 | 43 | 6.92 | 44 | 6.82 | 281 | 5.54 | 128 | 5.15 | 153 | 5.91 |
| Psoriasis | 53 | 4.18 | 28 | 4.51 | 25 | 3.87 | 235 | 4.63 | 157 | 6.32 | 78 | 3.01 |
| Primary adrenocortical insufficiency | 55 | 4.34 | 26 | 4.18 | 29 | 4.49 | 165 | 3.25 | 84 | 3.38 | 81 | 3.13 |
| Ankylosing spondylitis | 28 | 2.21 | 18 | 2.90 | 10 | 1.54 | 127 | 2.50 | 74 | 2.97 | 53 | 2.05 |
| Autoimmune encephalomyelitis | 32 | 2.52 | 16 | 2.57 | 16 | 2.47 | 32 | 0.63 | 18 | 0.72 | 14 | 0.54 |
| Rheumatoid arthritis | 13 | 1.02 | 5 | 0.80 | 8 | 1.24 | 69 | 1.36 | 11 | 0.44 | 58 | 2.24 |
| Sjögren's syndrome | 9 | 0.71 | ^b^ | ^b^ | ^b^ | ^b^ | 50 | 0.98 | ^b^ | ^b^ | ^b^ | ^b^ |
| Systemic lupus erythematosus | 6 | 0.47 | 3 | 0.48 | 3 | 0.46 | 11 | 0.22 | ^b^ | ^b^ | ^b^ | ^b^ |
| Myasthenia gravis | 5 | 0.39 | ^b^ | ^b^ | ^b^ | ^b^ | 17 | 0.33 | 9 | 0.36 | 8 | 0.31 |
| Guillain–Barré syndrome | 5 | 0.39 | ^b^ | ^b^ | ^b^ | ^b^ | 15 | 0.30 | 7 | 0.28 | 8 | 0.31 |

Abbreviation: IR, incidence rate per 10,000 person-years.

^b^ The cells left blank indicated that the number of events was less than three and were therefore not allowed to be exported under the regulations of the Health and Welfare Data Science Center of Taiwan to prevent re-identification.

*Data on Type I DM, systemic sclerosis, inflammatory myopathy, Behcet's syndrome, systemic vasculitis, multiple sclerosis, autoimmune hemolytic anemia, inflammatory myopathy, pemphigus, celiac disease, inflammatory bowel diseases and post-infectious arthritis cases were not shown because case numbers were less than three.

**Supplementary Table 3. Risk of autoimmune diseases among hospitalized dengue cases after adjusting for competing mortality.**

| Autoimmune disease | Total |  | Male |  | Female |  |
| --- | --- | --- | --- | --- | --- | --- |
|  | aHR^a^ (95% CI)^b^ | p value^c^ | aHR^a^ (95% CI)^b^ | p value^c^ | aHR^a^ (95% CI)^b^ | p value^c^ |
| All | 1.14 (1.01-1.28) | 0.0302 | 1.12 (0.94-1.34) | 0.1949 | 1.15 (0.98-1.34) | 0.0868 |
| Autoimmune thyroid disease | 1.15 (0.91-1.46) | 0.2352 | 1.41 (0.89-2.25) | 0.1428 | 1.08 (0.82-1.42) | 0.5935 |
| Uveitis | 1.14 (0.90-1.46) | 0.2805 | 1.20 (0.85-1.70) | 0.2928 | 1.08 (0.77-1.52) | 0.6586 |
| Psoriasis | 0.87 (0.64-1.18) | 0.3733 | 0.66 (0.44-1.00) | 0.0484 | 1.33 (0.83-2.14) | 0.2337 |
| Primary adrenocortical insufficiency | 1.33 (0.97-1.82) | 0.0794 | 1.30 (0.83-2.05) | 0.2571 | 1.36 (0.88-2.11) | 0.1642 |
| Ankylosing spondylitis | 0.88 (0.59-1.33) | 0.5445 | 1.02 (0.61-1.70) | 0.9476 | 0.73 (0.37-1.42) | 0.3489 |
| Autoimmune encephalomyelitis | 3.66 (2.19-6.14) | <0.0001 | 3.04 (1.53-6.03) | 0.0015 | 4.85 (2.18-10.80) | 0.0001 |
| Rheumatoid arthritis | 0.77 (0.42-1.40) | 0.3899 | 1.97 (0.62-6.30) | 0.2508 | 0.56 (0.26-1.17) | 0.1223 |
| Sjögren's syndrome | 0.76 (0.36-1.60) | 0.4719 | 2.93 (0.26-33.41) | 0.3863 | 0.70 (0.32-1.52) | 0.3669 |
| Systemic lupus erythematosus | 2.91 (1.05-8.09) | 0.0404 | 7.20 (0.94-54.95) | 0.0570 | 1.82 (0.49-6.75) | 0.3705 |
| Myasthenia gravis | 1.14 (0.42-3.09) | 0.7945 | 0.89 (0.20-3.88) | 0.8725 | 1.60 (0.40-6.37) | 0.5084 |
| Guillain–Barré syndrome | 1.60 (0.60-4.27) | 0.3480 | 1.42 (0.33-6.15) | 0.6403 | 1.95 (0.51-7.49) | 0.3314 |

Abbreviations: aHR, adjusted hazard ratio; CI, confidence interval.

^a^ Adjusted Cox proportional hazard model by sex, age, area of residence, income, urbanization, hypertension, diabetes mellitus, dyslipidemia, COPD, cerebrovascular accident, renal failure, liver cirrhosis, ischaemic heart disease, and malignancy.

^b^ 95% CIs were not adjusted for multiple comparisons and thus cannot be directly used for hypothesis testing or inference.

**Summary and General Comments**
Reviewer #1: As Above
Reviewer #2: This is a solid study. The authors should clarify that it remains possible that more severe dengue can lead to autoimmune disease.
**Response:** Please see the above response.

We hope this manuscript is ready for publication. Thank you very much for considering our manuscript submission.

Sincerely yours

Yu-Wen Chien

Department of Public Health, College of Medicine, National Cheng Kung University, Tainan, Taiwan

No. 1, University Road, Department of Public Health College of Medicine,

National Cheng Kung University, Tainan 70101, Taiwan

Tel: 886-6-2353535 ext. 5601

FAX: +886-6-2359033
